# Unraveling the Complexities of Hypersensitivity Pneumonitis with Autoimmune Features: A Retrospective Analysis

**DOI:** 10.3390/arm93060050

**Published:** 2025-11-07

**Authors:** Joana Lourenço, Sofia Castro, David Barros Coelho, André Terras Alexandre, Natália Melo, Patrícia Caetano Mota, Hélder Novais Bastos, André Carvalho, António Morais

**Affiliations:** 1 Pulmonology Department, Unidade Local de Saúde de Matosinhos, 4464-513 Porto, Portugal; 2 Pulmonology Department, Unidade Local de Saúde da Região de Aveiro, 3814-501 Aveiro, Portugal; sofiafcastro@hotmail.com; 3 Pulmonology Department, Unidade Local de Saúde de São João, 4200-319 Porto, Portugal; david.b.c@hotmail.com (D.B.C.); afntalexandre@gmail.com (A.T.A.); nataliafmelo@hotmail.com (N.M.); patcmota@gmail.com (P.C.M.); hnovaisbastos@gmail.com (H.N.B.); antonio.morais01@hotmail.com (A.M.); 4 Faculty of Medicine, University of Porto, 4200-319 Porto, Portugal; 5 Rise Health—Faculty of Medicine, University of Porto, 4200-319 Porto, Portugal; 6 Radiology Department, Unidade Local de Saúde de São João, 4200-319 Porto, Portugal; andre.silva.carvalho@chsj.min-saude.pt

**Keywords:** hypersensitivity pneumonitis, autoimmune features, immunosuppression, progressive pulmonary fibrosis

## Abstract

**Highlights:**

**What are the main findings?**

- Hypersensitivity pneumonitis with autoimmune features (HPAF) showed similar overall outcomes to classic hypersensitivity pneumonitis (HP) within the first two years after diagnosis.
- Under immunosuppressive therapy, HPAF patients developed significantly less progressive pulmonary fibrosis compared to HP group.

**What is the implication of the main finding?**

- The presence of autoimmune features may identify a subgroup of HP patients with a more favorable response to immunosuppressive therapy.
- These findings highlight the potential value of closer evaluation of autoimmune markers in HP to eventually guide therapeutic decisions.

**Abstract:**

Background: Some hypersensitivity pneumonitis (HP) patients exhibit autoimmune features (HPAF). This study compared outcomes of HPAF and HP without autoimmune features, focusing on progressive pulmonary fibrosis (PPF) and response to immunosuppression. Methods: A retrospective cohort study included HP patients from a single center. HPAF was defined as HP overlapping with autoimmune disease or presenting autoimmune markers/symptoms not fulfilling connective tissue disease criteria. A control HP group without autoimmune features was randomly selected. Demographics, autoimmune profiles, and outcomes over two years were analyzed. Results: 103 patients were included (52 HPAF; 51 HP). In HPAF, the most common autoimmune diseases were rheumatoid arthritis (9.6%), while 57.7% had isolated autoimmune serology. Groups showed no baseline differences in demographics, exposures, smoking, or lung function. Fibrotic disease on high-resolution CT at diagnosis was less frequent in HPAF (71.2% vs. 88.2%; *p* = 0.031). At two-year follow-up, survival, transplantation, and PPF prevalence were similar. HPAF patients received immunosuppression less often (69.2% vs. 86.3%; *p* = 0.038). Among patients under immunosuppression, PPF was significantly lower in HPAF group (8.6% vs. 29.5%; *p* = 0.021). Conclusions: Within two years post-diagnosis, HPAF and HP had comparable overall outcomes. However, under immunosuppression, HPAF patients had significantly lower odds of developing PPF (adjusted OR 0.08; 95% CI 0.008–0.816; *p* = 0.033) compared to HP patients, suggesting a more favorable treatment response.

## 1. Introduction

The relationship between connective tissue disease (CTD) and various forms of interstitial lung disease (ILD) is well established [1]. Additionally, the recognition of autoimmune features in idiopathic interstitial pneumonias has led to the definition and classification of interstitial pneumonia with autoimmune features (IPAF) [2,3].

Similarly, published studies have identified hypersensitivity pneumonitis (HP) patients with coexisting positive autoantibodies, systemic inflammatory features, or overlapping autoimmune diseases [4,5]. Data on the prevalence of HP with such autoimmune features (HPAF) remain limited. However, Adegunsoye et al. reported that, within a cohort of fibrotic HP patients, approximately 15% met the criteria for HPAF [4].

Currently, pharmacological treatment strategies for HPAF are similar to those used for HP, typically involving a combination of immunosuppressive agents (such as corticosteroids, immunomodulators, and biologic therapies) and antifibrotics, tailored according to disease severity and individual patient characteristics [5,6].

The pathogenesis of HPAF remains unclear and continues to represent a controversial and poorly understood entity [7]. Environmental antigen diversity has been shown to impact disease expression and prognosis in HP [8]. These environmental exposures may trigger an initial inflammatory cascade, leading to activation of autoreactive immune cells and the production of autoantibodies against self-antigens in genetically susceptible individuals [9,10]. This complex interplay between environmental triggers and autoimmune dysregulation underscores the heterogeneous nature of HP and highlights the need for personalized diagnostic and therapeutic approaches [11].

From a prognostic perspective, it is also uncertain whether this autoimmune basis confers a better outcome for HPAF patients, similar to cases of CTD-ILD, particularly when treated with immunosuppressive therapy. The aim of this article is to investigate this hypothesis by comparing HPAF and HP without autoimmunity in terms of disease progression.

## 2. Materials and Methods

All HPAF outpatients followed at the Unidade Local de Saúde de São João, a university referral hospital in Portugal, until 2023 were included. Patients were identified through electronic medical records, comprising individuals referred between 2009 and 2023. Data were collected between May and December 2023. Patients were required to fulfill the 2022 ATS/JRS/ALAT diagnostic criteria for HP [12]. HPAF was defined as HP overlapping with autoimmune diseases or presenting with isolated positive autoimmune features (serological or clinical), without meeting formal diagnostic criteria for CTD. Specific CTD-related symptoms considered included arthralgia, joint swelling, morning stiffness, sicca symptoms, proximal muscle weakness, or Raynaud’s phenomenon [4]. Positive autoimmune serologies included: anti-nuclear antibody (ANA) at titers ≥1:320 (or any titer in cases showing ANA nucleolar or centromere patterns); rheumatoid factor (RF) ≥ three times the upper limit of normal; reduced complement levels; or positive anti-neutrophil cytoplasmic antibodies (ANCA), cyclic citrullinated peptide (CCP), Jo1, dsDNA, nucleosome, RNP, Smith (SM), Scl-70, SS-A/Ro, or SS-B/La antibodies [4,13,14,15]. Serological analyses were conducted at the hospital’s accredited Immunology Laboratory. Standardized commercial assays routinely used in clinical practice were employed, including enzyme-linked immunosorbent assays (ELISAs), indirect immunofluorescence, and standardized automated immunoassays.

A control group of HP patients without autoimmune features, followed at the same center, was randomly selected. All diagnoses and patients were reviewed at multidisciplinary team meetings comprising pulmonologists, radiologists, and pathologists with expertise in ILD. 

The research was conducted according to the principles of the World Medical Association´s Declaration of Helsinki. Written informed consent was obtained from all participants.

A retrospective comparison between the two groups was performed, focusing on demographics, serological autoimmune profiles or autoimmune diagnoses, lung function parameters, high-resolution chest CT (HRCT), bronchoalveolar lavage (BAL) characteristics, and clinical outcomes within the first two years after diagnosis. Progressive fibrosis was defined according to the 2022 ERS/ATS criteria for progressive pulmonary fibrosis (PPF) [16].

Statistical analysis was conducted using IBM SPSS Statistics (version 23), with a *p*-value < 0.05 considered statistically significant. Missing data were handled using pairwise deletion. 

## 3. Results

A total of 103 patients were enrolled: 52 in the HPAF group and 51 in the HP control group. In the HPAF group, the most frequent overlapping autoimmune diseases were rheumatoid arthritis (RA) in 9.6% and Sjögren’s syndrome in 7.7%. Nonetheless, in this sample, most individuals (57.7%) exhibited only a positive autoimmune serological profile without clinical symptoms suggestive of CTD, with ANA and RF being the most commonly positive parameters (86.5% and 25.0%, respectively). 

No significant differences were observed between the groups regarding baseline characteristics, including age, gender, environmental exposures, smoking habits, and lung function parameters—namely, forced vital capacity (FVC) and carbon monoxide diffusing capacity (DLCO)—as represented in Table 1. However, at baseline HRCT, the presence of fibrotic disease was significantly lower in the HPAF group (HPAF 71.2% vs. HP 88.2%; *p* = 0.031). 

Table 2 reveals the overall follow-up variables analyzed between the two groups. There were no statistically significant differences in the number of deaths (HPAF 17.3% vs. HP 15.7%; *p* = 0.825) or lung transplants (HPAF 0% vs. HP 2.0%; *p* = 0.541). Likewise, Kaplan–Meier survival analysis showed no significant differences (Log-rank = 0.901), with a median survival of 9.4 years (CI 8.0–10.8) for the HPAF group versus 9.6 years (CI 8.1–11.0) for the HP group. The overall prevalence of PPF during follow-up was also similar between groups (HPAF 37.3% vs. HP 43.1%; *p* = 0.545).

### 3.1. HP Under Immunosuppressive Therapy

Over the two-year follow-up period after diagnosis, the HPAF group was significantly less exposed to immunosuppressive treatment (HPAF 69.2% vs. HP 86.3%; *p* = 0.038). The other patients (n = 23, of whom 7 from the HP control group and 16 from the HPAF group) remained under surveillance during the follow-up period, primarily through antigen avoidance.

A subgroup analysis was performed among patients receiving immunosuppressive therapy. Table 3 presents the baseline characteristics, which were similar for both subgroups, including fibrotic features on initial HRCT and pulmonary function tests. After diagnosis, the HPAF group initiated immunosuppressive therapy significantly later: median 107.0 days (range 7.5–343.5) versus 56.0 days (range 7.0–109.0) in the HP group (*p* = 0.040). The most commonly used drugs were systemic corticosteroids (HPAF n = 32 vs. HP n = 23), mycophenolate mofetil (HPAF n = 29 vs. HP n = 41), and azathioprine (HPAF n = 10 vs. HP n = 10). It should be noted that some patients were exposed to more than one immunosuppressive agent during the study follow-up period.

Table 4 displays the variables analyzed between the two subgroups during follow-up. No significant differences were observed regarding hospital admissions for ILD exacerbation, home supplementary oxygen therapy, lung transplantation, number of deaths, or overall survival after diagnosis. However, the proportion fulfilling criteria for PPF within the two-year follow-up was significantly lower in the HPAF group (HPAF 8.6% vs. HP 29.5%; *p* = 0.021).

To identify factors significantly associated with the development of PPF among patients treated with immunosuppressive agents, a logistic regression analysis was performed. Table 5 presents the multivariable analysis model. After adjustment for gender, age, smoking status, baseline FVC and DLCO, the presence of fibrotic disease at diagnosis and the length of immunosuppressive therapy during the two-year follow-up period, the HPAF group remained associated with a significantly lower risk of PPF (adjusted OR 0.080; 95%CI: 0.008–0.816; *p*-value 0.033).

### 3.2. HP with Fibrotic Features at Diagnosis

A subgroup analysis of patients presenting with fibrotic features at diagnosis (HPAF 71.2%; HP 88.2%) was conducted. No significant differences were found in the baseline characteristics between the two groups, including age, gender, environmental exposures, smoking habits, and lung function (FVC and DLCO).

During the two-year follow-up period, both groups had similar exposure to immunosuppressive therapy (HPAF 75.7%, HP 88.9%; *p* = 0.114). Only one patient from each group initiated antifibrotic therapy (nintedanib). Table 6 summarizes the follow-up variables analysed between the two groups. No significant differences were observed in terms of hospital admissions for ILD exacerbation, use of home supplementary oxygen, fulfilling criteria for PPF, lung transplantation, mortality, or overall survival after diagnosis.

## 4. Discussion

It could be hypothesized that the presence of autoimmune features might confer a better prognosis in patients with HP, particularly those receiving immunosuppressive therapy. However, the currently available evidence regarding HPAF is inconsistent. Adegunsoye et al. concluded that the presence of autoimmune features or an autoimmune disease independently predicted worse outcomes among patients with fibrotic HP [4]. In contrast, Kalra and colleagues found that, although HPAF patients had a higher probability of developing pulmonary hypertension, they did not have a poorer overall prognosis compared to patients with non-HPAF HP [5].

In our study, patients with HPAF showed a significantly lower prevalence of fibrotic disease at presentation (71.2% vs. 88.2%; *p* = 0.031), suggesting a milder form of HP at diagnosis. Additionally, during the initial two-year follow-up, the HPAF group was significantly less likely to receive immunosuppressive therapy (HPAF 69.2% vs. HP 86.3%; *p* = 0.038), and such therapy was initiated significantly later after diagnosis [HPAF: 107.0 days (7.5–343.5) vs. HP: 56.0 days (7.0–109.0); *p* = 0.040]. This may reflect a milder disease course in the HPAF group, reducing the need for early targeted treatment. 

Surprisingly, in our sample, there were no significant differences in the number of lung transplantations, deaths, or overall survival. These results are consistent with the findings of Kalra et al., who reported that patients with HPAF had a prognosis similar to those with HP [5]. However, an earlier study on HPAF suggested a potentially worse prognosis in these patients [4]. This discrepancy may be explained by the fact that different studies enrolled HPAF patients based on varying definitions, reflecting the lack of consensus on this entity. As a result, comparative analysis between the few available studies is challenging, leading to inconsistent findings regarding HPAF prognosis. In our study, patients were classified as HPAF using a broader definition. As more than half (57.7%) of our HPAF cohort had only positive serology in the absence of clinical autoimmune manifestations, the lack of significant prognostic differences between the groups in the first two years after diagnosis becomes more understandable. This heterogeneity in the inclusion criteria for the HPAF group likely reduced potential prognostic contrasts. 

As in other ILDs, cases of HP show a well-described association between progressive fibrotic behavior and poor prognosis [17,18]. In this sample, among patients with fibrotic disease at diagnosis, no significant differences were found in baseline characteristics or prognostic variables between the HPAF and HP groups.

When a subgroup analysis was performed, including only patients from each group who had initiated immunosuppressive therapy, a significantly lower prevalence of PPF (8.6% vs. 29.5%) was observed in the HPAF group. These findings suggest that the presence of autoimmune characteristics may confer a more favourable response to immunosuppressive treatment, despite overall survival being comparable in this sub-analysis. It could be argued that the lower extent of fibrotic disease at diagnosis and the later initiation of immunosuppressive therapy in the HPAF group might have introduced bias in data interpretation. To address this limitation, a multivariable logistic regression analysis was performed. Even after adjustment for gender, age, smoking status, baseline pulmonary function, fibrotic disease at diagnosis, and length of immunosuppressive therapy during follow-up, patients in the HPAF group receiving immunosuppressive therapy had a significantly lower likelihood of developing PPF comparing to HP patients exposed to the same treatment (adjusted OR 0.08; 95% CI 0.008–0.816; *p* = 0.033). This may indeed suggest a better therapeutic response in patients with HPAF.

To our knowledge, this study presents one of the largest cohorts of HPAF ever published, enabling a comprehensive characterization of these patients and comparison with a control HP group over a substantially extended period following diagnosis.

In terms of limitations, the first relates to the retrospective design of the study. Second, our cohorts were drawn from a single tertiary referral center with expertise in ILD/CTD, and, therefore, our findings require external validation. Additionally, although this is one of the larger HPAF cohorts analysed, the sample size remains relatively small.

In summary, HPAF represents a complex and evolving entity at the intersection of environmental hypersensitivity reactions and autoimmune diseases. Further research is needed to clarify the definition of HPAF, and the true implications of autoimmunity on patient outcomes must be elucidated to guide therapeutic decisions.

## 5. Conclusions

In this study, no differences were found in major outcomes between HPAF and HP patients during the first two years after diagnosis. A subgroup analysis of patients receiving immunosuppressive therapy revealed that the prevalence of PPF was significantly lower in the HPAF group during this period, suggesting a possible trend toward a more favorable response to immunosuppressive treatment in these patients. Further studies are warranted to confirm and better understand these potential differences in treatment response.

## Figures and Tables

**Table 1:** Baseline characteristics of HP and HPAF groups.

Baseline Characteristics	HPAF Group (n = 52)	HP Control Group (n = 51)	*p*-Value
Age (years), mean (±SD)	74.0 ± 9.8	70.4 ± 8.1	0.163
Female gender, n (%)	31 (59.6%)	28 (54.9%)	0.629
Caucasian, n (%)	52 (100.0%)	51 (100.0%)	-
Smoking habits, n (%)			0.511
Active smoker	0 (0%)	1 (2.0%)	
Ex-smoker	17 (32.7%)	19 (37.3%)	
Non-smoker	35 (67.3%)	31 (60.8%)	
Pack-Year smoked, mean (±SD)	38.1 ± 24.6	32.5 ± 17.2	0.441
Environmental exposure, n (%)			
Avian	42 (80.8%)	45 (88.2%)	0.296
Mold	14 (26.9%)	22 (43.1%)	0.084
Paints	1 (1.9%)	5 (9.8%)	0.112
Others (jacuzzi, dust or feathers)	17 (32.7%)	9 (17.6%)	0.079
No exposure identified	2 (3.8%)	1 (2.0%)	0.569
Family history of ILD, n (%)	2 (3.8%)	2 (3.9%)	0.984
Fibrotic HP at initial HRCT, n (%)	37 (71.2%)	45 (88.2%)	0.031 *
Baseline PFT			
FVC (L), mean (±SD)	2.3 ± 0.8	2.3 ± 0.8	0.800
FVC (% of predicted), mean (±SD)	80.68 ± 17.11	78.67 ± 17.97	0.621
DLCO (mmol∙min^−1^∙kPa^−1^), mean (±SD)	4.0 ± 1.3	3.8 ± 1.3	0.385
DLCO (% of predicted), mean (±SD)	54.90 ± 16.17	51.13 ± 16.36	0.264
BAL, n (%)	49 (94.2%)	44 (86.3%)	0.201
BAL lymphocytes (%), median (Q1–Q3)	25.5 (13.2–47.9)	24.1 (12.9–39.9)	0.796

Absolute frequency (n); absolute percentage (%); Standard deviation (SD); Percentage of predicted value (% of predicted); Pulmonary function test (PFT); Forced vital capacity (FVC); Carbon monoxide diffusing capacity (DLCO); High-resolution CT (HRCT); Bronchoalveolar lavage (BAL); Statistical significance (*).

**Table 2:** HP and HPAF groups characterization within 2-year follow-up.

Characterization Within 2-Year Follow-Up	HPAF Group (n = 52)	HP Control Group (n = 51)	*p*-Value	OR (95% CI)
PPF criteria (ERS/ATS 2022), n (%)	19 (37.3%)	22 (43.1%)	0.545	0.783 (0.354–1.730)
Lung transplant referral, n (%)	3 (5.8%)	5 (9.8%)	0.488	0.563 (0.127–2.491)
Death, n (%)	9 (17.3%)	8 (15.7%)	0.825	1.125 (0.397–3.189)
Survival after diagnosis (years), Kaplan–Meier analysis	9.41 (95% CI 8.01–10.81)	9.58 (95% CI 8.09–11.07)	0.901	-

Absolute frequency (n); absolute percentage (%); odds ratio (OR); confidence interval (95% CI); Progressive Pulmonary Fibrosis (PPF).

**Table 3:** Baseline characteristics of HP and HPAF subgroups treated with immunosuppressive agents.

Baseline Characteristics	HPAF Group (n = 36, 69.2%)	HP Control Group (n = 44, 86.3%)	*p*-Value
Age (years), mean (±SD)	70.5 ± 11.2	70.2 ± 8.4	0.315
Female gender, n (%)	22 (61.1%)	25 (56.8%)	0.698
Smoking habits, n (%)			0.629
Active smoker	0 (0%)	1 (2.3%)	
Ex-smoker	13 (36.1%)	17 (38.6%)	
Non-smoker	23 (63.9%)	26 (59.1%)	
Environmental exposure, n (%)			
Avian	30 (83.3%)	38 (86.4%)	0.706
Mold	10 (27.8%)	20 (45.5%)	0.104
Fibrotic HP at initial HRCT	28 (77.8%)	40 (90.9%)	0.102
Baseline PFT			
FVC (L), mean (±SD)	2.3 ± 0.7	2.3 ± 0.8	0.799
FVC (% of predicted), mean (±SD)	78.4 ± 17.7	77.7 ±18.3	0.864
DLCO (mmol∙min^−1^∙kPa^−1^), mean (±SD)	3.9 ± 1.1	3.7 ± 1.3	0.642
DLCO (% of predicted), mean (±SD)	52.0 ± 15.3	49.2 ± 16.5	0.455

Absolute frequency (n); absolute percentage (%); Percentage of predicted value (% of predicted); Standard deviation (SD); Pulmonary function test (PFT); Forced vital capacity (FVC); Carbon monoxide diffusing capacity (DLCO); High-resolution CT (HRCT).

**Table 4:** Subgroup of HP and HPAF patients under immunosuppressive therapy, characterization within 2-year follow-up.

Characterization Within 2-Year Follow-Up	HPAF Group (n = 36, 69.2%)	HP Control Group (n = 44, 86.3%)	*p*-Value	OR (95% CI)
Hospitalization for ILD exacerbation, n (%)	7 (19.4%)	8 (18.2%)	0.886	1.086 (0.352–3.349)
Supplementary oxygen home therapy, n (%)	8 (22.2%)	8 (18.2%)	0.653	1.286 (0.429–3.852)
PPF criteria (ERS/ATS 2022), n (%)	3 (8.3%)	13 (29.5%)	0.021 *	0.224 (0.058–0.862)
Lung transplant referral, n (%)	3 (8.3%)	5 (11.4%)	0.851	0.709 (0.158–3.192)
Death, n (%)	8 (22.2%)	8 (18.2%)	0.653	1.286 (0.429–3.852)
Survival after diagnosis (years), Kaplan–Meier analysis	9.11 (95% CI 7.48–10.75)	8.89 (95% CI 7.04–10.74)	0.857	-

Absolute frequency (n); absolute percentage (%); odds ratio (OR); confidence interval (95% CI); interstitial lung disease (ILD); Progressive Pulmonary Fibrosis (PPF); Statistical significance (*).

**Table 5:** Multivariable analysis in Logistic regression for PPF predictors in patients under immunosuppressive therapy.

Variable	*p*-Value	Adjusted OR (95% CI)
HPAF group	0.033 *	0.080 (0.008–0.816)
Female gender	0.935	1.076 (0.182–6.361)
Age (years)	0.019 *	1.169 (1.026–1.333)
Ever smoker	0.662	1.461 (0.268–7.970)
Baseline FVC (% of predicted)	0.902	0.997 (0.946–1.050)
Baseline DLCO (% of predicted)	0.798	1.008 (0.951–1.067)
Fibrotic HP at initial HRCT	0.363	2.628 (0.328–21.076)
Time exposed to immunosuppressive therapy (days)	0.383	0.998 (0.994–1.002)

Percentage of predicted value (% of predicted); Forced vital capacity (FVC); Carbon monoxide diffusing capacity (DLCO); High-resolution CT (HRCT); odds ratio (OR); confidence interval (95% CI); Statistical significance (*).

**Table 6:** Subgroup of HP and HPAF patients presenting fibrotic features at diagnosis, characterization within 2-year follow-up.

Characterization Within 2-Year Follow-Up	HPAF Group (n = 37, 71.2%)	HP Control Group (n = 45, 88.2%)	*p*-Value	OR (95% CI)
Hospitalization for ILD exacerbation, n (%)	7 (18.9%)	8 (17.8%)	0.894	1.079 (0.351–3.317)
Supplementary oxygen home therapy, n (%)	7 (18.9%)	8 (17.8%)	0.894	1.079 (0.351–3.317)
PPF criteria (ERS/ATS 2022), n (%)	16 (35.6%)	13 (35.1%)	0.968	0.982 (0.395–2.439)
Lung transplant referral, n (%)	1 (2.7%)	5 (11.1%)	0.324	0.222 (0.025–1.993)
Death, n (%)	8 (21.6%)	7 (15.6%)	0.480	1.498 (0.487–4.606)
Survival after diagnosis (years), Kaplan–Meier analysis	9.31 (95% CI 7.80–10.81)	8.07 (95% CI 6.87–9.28)	0.589	-

Absolute frequency (n); absolute percentage (%); odds ratio (OR); confidence interval (95% CI); interstitial lung disease (ILD); Progressive Pulmonary Fibrosis (PPF).

## Data Availability

Data are contained within the article.

## Abbreviations

The following abbreviations are used in this manuscript:

ANA	Anti-nuclear antibody
ANCAs	Anti-neutrophil cytoplasmic antibodies
BAL	Bronchoalveolar lavage
CCP	Cyclic citrullinated peptide
CTD	Connective tissue disease
DLCO	Carbon monoxide diffusing capacity
FVC	Forced vital capacity
HP	Hypersensitivity pneumonitis
HPAF	Hypersensitivity pneumonitis autoimmune feature
HRCT	High-resolution chest CT
ILD	Interstitial lung disease
IPAF	Interstitial pneumonia with autoimmune features
PPF	Progressive pulmonary fibrosis
RA	Rheumatoid arthritis
RF	Rheumatoid factor
